# Bio-inspired Plasmonic Nanoarchitectured Hybrid System Towards Enhanced Far Red-to-Near Infrared Solar Photocatalysis

**DOI:** 10.1038/srep20001

**Published:** 2016-01-28

**Authors:** Runyu Yan, Min Chen, Han Zhou, Tian Liu, Xingwei Tang, Ke Zhang, Hanxing Zhu, Jinhua Ye, Di Zhang, Tongxiang Fan

**Affiliations:** 1 State Key Lab of Metal Matrix Composites, Department of Materials Science and Engineering, Shanghai Jiaotong University, Shanghai, 200240, China; 2 International Center for Materials Nanoarchitectonics (WPI-MANA) and Environmental Remediation Materials Unit, National Institute for Materials Science (NIMS), 1-1, Namiki, Tsukuba, Ibaraki 305-0044, Japan; 3 TU−NIMS Joint Research Center, School of Materials Science and Engineering, Tianjin University, 92 Weijin Road, Nankai District, Tianjin 300072, P. R. China; 4 School of Engineering, Cardiff University, Cardiff, CF24 3AA, UK

## Abstract

Solar conversion to fuels or to electricity in semiconductors using far red-to-near infrared (NIR) light, which accounts for about 40% of solar energy, is highly significant. One main challenge is the development of novel strategies for activity promotion and new basic mechanisms for NIR response. Mother Nature has evolved to smartly capture far red-to-NIR light via their intelligent systems due to unique micro/nanoarchitectures, thus motivating us for biomimetic design. Here we report the first demonstration of a new strategy, based on adopting nature’s far red-to-NIR responsive architectures for an efficient bio-inspired photocatalytic system. The system is constructed by controlled assembly of light-harvesting plasmonic nanoantennas onto a typical photocatalytic unit with butterfly wings’ 3D micro/nanoarchitectures. Experiments and finite-difference time-domain (FDTD) simulations demonstrate the structural effects on obvious far red-to-NIR photocatalysis enhancement, which originates from (1) Enhancing far red-to-NIR (700~1200 nm) harvesting, up to 25%. (2) Enhancing electric-field amplitude of localized surface plasmon (LSPs) to more than 3.5 times than that of the non-structured one, which promotes the rate of electron-hole pair formation, thus substantially reinforcing photocatalysis. This proof-of-concept study provides a new methodology for NIR photocatalysis and would potentially guide future conceptually new NIR responsive system designs.

Solar conversion to fuels or to electricity in semiconductors is a highly significant and challenging scientific and commercial mission^1^,^2^,^3^. Up to now, most efforts are focused on UV and visible light photocatalysis^4^,^5^,^6^ while near-infrared (NIR) light which accounts for about 40% of the solar energy is rarely utilized but quite important. So far, however, the problems are limited NIR active photocatalysts (e.g. Cu_2_(OH)PO_4_^7^, WS_2_^8^, BiErWO_6_^9^, defect-induced Bi_2_WO_6_^10^, H-WO_3_^11^, H-TiO_2_^12^, etc.), low efficiency, and restricted mechanisms (e.g. upconversion process^13^, carbon quantum dots doping^14^, heterostructure design^15^,^16^, oxygen vacancies induction^11^,^12^, localized surface plasmon resonance nanoparticles^17^,^18^ modification etc.). One main challenge towards NIR photocatalysis therefore is the development of novel design pathways for enhanced activity and new basic principles for NIR response, which would open a promising direction for the field of NIR photocatalysis. Biomimetics is a splendiferous strategy for scientific and technological problems^19^,^20^,^21^,^22^. Mother Nature has already known to smartly use the wide spectrum and quantum rich solar irradiation field (far red-to-NIR light) via their evolved intelligent systems with superior components and elaborated architectures. In photosynthesis, a water-oxidizing cyanobacteria has evolved to use 700~740 nm light while bacterial reaction centers are able to utilize photons as ‘red’ as 1030 nm^23^. Some pioneering endeavors discovered that certain biological systems (e.g. snake skins^24^,^25^, butterfly wings^26^) are able for NIR harvesting due to their unique micro/nanoarchitectures with underlying mechanisms. However, so far as we know, these NIR harvesting mechanisms in biology have never been applied for the design of new efficient NIR responsive systems. Here we report a new strategy, based on adopting nature’s NIR light responsive architectures prototypes for an efficient far red-to-NIR photocatalytic system. This research would potentially lead to an entirely new paradigm in harvesting NIR photons for practical use.

Natural systems have evolved various optical properties (e.g. structural color, circular polarization, iridescence, color mixing, etc.) because of their unique structures (e.g. multilayer, helicoidal structure, photonic crystals, double-facet microlens and so forth)^27^. In particular, certain biological unique architectures are discovered to result in enhanced NIR responsive activity with underlying mechanisms. Black scales on certain snake skins and unique infrared imaging pit organs of boid snakes were reported to promote NIR absorbance due to their hierarchical pattern of leaf-like microstructures with nanoridges^24^ and arrays of pore-like structures^25^, respectively. Aside from snakes, some pioneering work discovered that certain colored scales of butterfly wings are able for NIR harvesting due to their photonic crystals^26^. Furthermore, inspired by nano-architectures of iridescent *Morpho* butterfly scales, a highly sensitive infrared detector was demonstrated^28^ which can efficiently upconvert mid-infrared radiation to visible iridescence changes. Thus, the exploration and understanding of the NIR responsive mechanisms and further integration of bio-inspired 3D architectures with NIR-active photocatalysts for biomimetic design are highly appealing.

Plasmonic metallic nanostructures (*e.g.* Au nanorods/nanocages/nanospheres, Ag nanowires/nanocubes, Cu nanoparticles, etc) can serve as antennas to absorb visible-to-NIR light through localized surface plasmon resonance (LSPR)^18^,^29^,^30^, which has emerged as promising areas in nanophotonics, photocatalysis, photovoltaics, photodetectors, etc. The LSPR-induced photoactive properties have been ascribed to three possible mechanisms: (1) Charge injection^18^,^31^,^32^. The metallic nanostructures act as a sensitizer, absorbing light, exciting hot electrons and injecting them into the conduction band of the semiconductor. (2) Near-field electromagnetic enhancement^33^,^34^, which can enlarge the absorption of light up to orders of magnitude and promote the generation and separation rates of electron-hole pairs. (3) Synergistic effects of charge injection and near-field electromagnetic enhancement^35^. Incorporating plasmonic metal nanostructures with semiconductors in different forms such as semiconductors decorated with plasmonic metals, plasmonic metal/semiconductor core-shell or shell-core nanostructures, Janus (plasmonic metal)/semiconductor nanostructures and so forth has recently been widely studied for enhancing photocatalytic activity. However, to the best of our knowledge, few studies have investigated the synergistic effects of plasmonic metal nanostructures and semiconductors with unique 3D micro/nanoarchitectures on light harvesting and LSPR-induced electric field. Fortunately, the large variety of biological systems suggests that integrating optical frequency antenna nanostructures with biological elaborated architectures may provide opportunities to investigate their light-matter interactions.

Motivated thus, we propose a new strategy, inspired by nature’s NIR light responsive architectures prototypes for an efficient far red-to-NIR plasmonic hybrid photocatalytic system (Fig. 1). The system is based on controlled assembly of light-harvesting plasmonic nanoantennas (gold nanorods-Au NRs) onto a typical photocatalytic unit (bismuth vanadate) with biological 3D architecture designs. Four typical architectures of butterfly wing scales are discovered here to have enhanced far red-to-NIR light harvesting ability and computational simulation explains the origin of the response property. Experimental and finite-difference time-domain (FDTD) simulations are performed to obtain insight into the mechanisms for structural effects: (1) Enhancing far red-to-NIR light (700~1200 nm) harvesting ability, up to 25% enhancement. (2) Enhancing the electric field (E-field) amplitude of LSPs due to the coupling of the LSPs with the 3D architectures of the butterfly wings, with the electric-field intensity amplified to more than 3.5 times than the non-structured one. Photocatalytic gaseous isopropyl alcohol (IPA) degradation, as a model reaction, is conducted under far red-to-NIR irradiation to further demonstrate the structural effect. This study provides a new methodology for NIR photocatalysis *via* morphology control, particularly inspired from nature’s biological architecture designs. This is also a pioneering work on the light-matter interaction between plasmonic metal nanostructures and semiconductors with unique 3D micro/nanoarchitectures. The research would guide future conceptually new NIR responsive system designs.

## Results

### Morphology Characterization of Butterfly Wings

Butterfly wings are selected as architectures models for this proof-of-concept study because of their large variety and species-specific. They have evolved various optical functions such as structural colors^36^, circular polarization^37^, dynamic color change^38^, and so forth because of their 3D unique architectures. Here scales of *Papilio nireus* (Fig. 2a1–a3), *Papilio paris* (Fig. 2b1–b3), *Troides helena*’s forewing (Fig. 2c1–c3) and *Troides helena*’s underwing (Fig. 2d1–d3) are chosen as prototype models to focus on the optical absorption function. In general, each kind of microstructures has common characteristics: (1) periodic inverse V-type ridges parallel to the longitudinal axis of the scale; (2) a basal substrate; (3) an air space with disordered columnar pillars as upholders above the substrate (except *Troides helena*’s underwing). In term of distinctions, in the case of *Papilio nireus* (Fig. 2a2, a3), a set of hole arrays distribute hexagonally in the host lamina, which form a 2D photonic crystal (2D PC). The basal substrate consists of two chitin layers and a chitin-air layer between them, which forms a Bragg reflector (DBR)^39^; The rectangular nano-hole arrays in *Papilio paris* (Fig. 2b2, b3) and *Troides helena*’s forewing (Fig. 2c2, c3) appear to be double-row and triple-row patterned respectively; *Troides helena*’s underwing (Fig. 2d2, d3) only possesses ridges combined with basal substrate. Based on the morphology characterization, 3D schematic models (Fig. 2a4-d4) are established for further theoretical optical simulations. Specifically, all pillars, which have tiny contributions to optical absorption are neglected^26^.

### Optical Simulations on Far Red-to-NIR Harvesting

**Figure 1:**
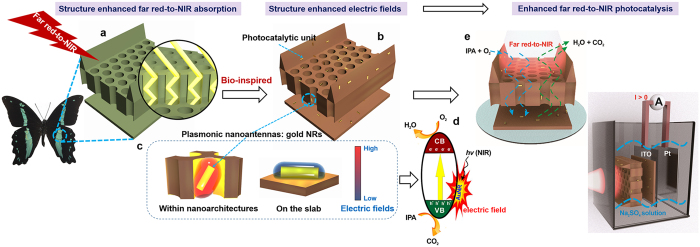
Schematic illustration of the concept of structure-enhanced bio-inspired far red-to-NIR highly responsive photocatalytic system. (**a**) The microstructural model from butterfly wing scales Papilio nireus revealing the far red-to-NIR absorption enhancement due to the 3D architectures. (**b**) light-harvesting plasmonic nanoantennas (gold NRs) onto a typical photocatalytic unit (bismuth vanadate) with biological 3D architectures. (**c**) Illustration of structure-enhanced LSPR-induced electric fields by the comparison between Au NRs within nanoarchitectures and Au NRs on the slab. (**d**) Illustration of the mechanism of isopropyl alcohol (IPA) photodegradation. (**e**) Photocatalytic processes for IPA degradation and photoelectrocatalytic process.

**Figure 2:**
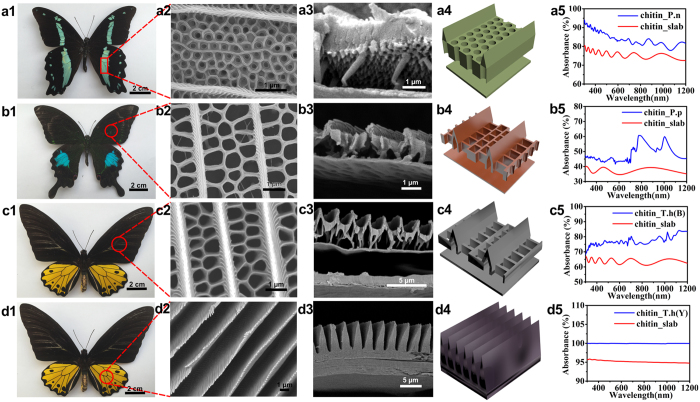
Overall views, morphologies, models and optical properties of original butterfly wing scales. (**a1,b1,c1,d1**) Digital photographs, (**a2,b2,c2,d2**) SEM images of top view, (**a3,b3,c3,d3**) SEM images of sectional view, (**a4,b4,c4,d4**) Simulation models and (**a5,b5,c5,d5**) Simulative optical absorption vs wavelength curves of butterfly wings (*Papilio nireus, Papilio paris, Troides helena*’s forewing and *Troides helena*’s underwing, respectively). The slab model was established by squeezing the corresponding structural models into a rectangle slab with equal length, width and volume.

Optical simulation was conducted by FDTD method based on the models extracted above with specific parameters taken from FESEM images (Supplementary Table S1). Non-structural slab model was constructed as the contrast sample (see Methods). Figure 2a5–d5 present the simulation results on optical absorption of the original model (blue line) and the planar slab model (red line). Generally, compared with the slab model, the absorption enhancement at far red-to-NIR wavelengths (700~1200 nm) reaches about 12%, 25%, 15% and 5% respectively. The corresponding experimental optical absorption further confirms the absorption reinforcement (Supplementary Fig. S1). The mechanism of absorption enhancement are mainly based on Multi-reflection theory^40^ and effective RI (refractive index) theory^41^. (1) Multi-reflection theory. The architectures interpreted by this theory are characterized by hole arrays, typically in Fig. 2a4–c4. Incident light illuminates into the holes, where multi-reflection occurs, then incident photons are largely absorbed during the multi-reflection process. Intriguingly, in Fig. 2b5, an extraordinary absorption peak appears, which might be caused by the resonance between holes and the incident light^40^. (2) Effective RI theory. This is applicable to the *Troides helena*’s underwing model shown in Fig. 2d4. From the top down, the effective RI of the inverse V-type ridges gradually increases from RI_air_ (1.0) to RI_chitin_ (1.56 + 0.06i), which avoids the dramatic change of refractive index, leading to absorption enhancement^41^. Therefore, it stands to reason that the theories apply to all butterfly wing structures characterized by hole arrays and inverse V-type ridges. Hence though only four kinds of scales are studied here, they can represent the dominant way of structure-enhanced absorption (far red-to-NIR region) in butterfly wings. Herein, as the light-harvesting plasmonic nanoantennas (gold nanorods) are sporadically loaded on the ingredient material, the Multi-reflection theory is more likely to help. Since the light absorption of *Papilio nireus* scales (85%) is superior to that of the other two (55% of *Papilio paris* scales and 80% of *Troides helena*’s forewing scales), the typical architecture of *Papilio nireus* scales was chosen as a prototype architecture for the further NIR photocatalysis realization (Fig. 1a).

### Development of bio-inspired far red-to-NIR photocatalytic system

**Figure 3:**
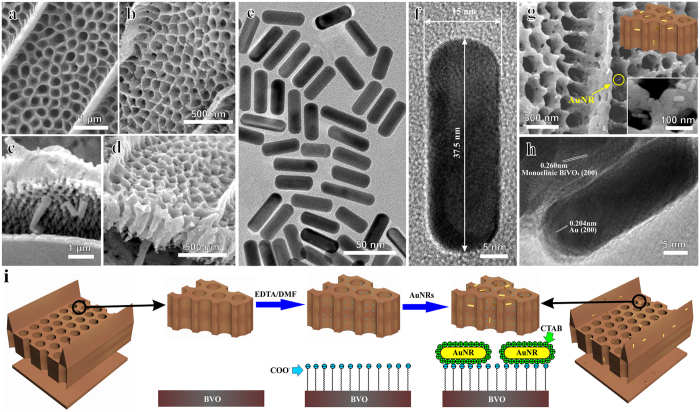
Morphology characterizations and schematic illustration for the synthetic procedures. FESEM images of (**a**) top view of original scale, (**b**) top view of BVO wing, (**c**) cross-section of original scale (**d**) cross-section of BVO wing. (**e**) TEM image of the Au NRs. (**f**) HRTEM image of a single Au NR. (**g**) Au NRs-loaded BVO wing with the insets of the simplified model (up) and amplified image (down). (**h**) HRTEM image of Au NRs-loaded BVO wing. (**i**) Schematic diagram of the controlled assembly of Au NRs onto BVO unit.

Bismuth vanadate (BVO) was chosen as a typical photocatalytic unit here because it is a widely investigated visible light responsive material^42^,^43^ for water splitting, photoelectrochemistry, photodegradation, etc. Pure Monoclinic BVO wing was synthesized *via* a sol-gel method derived from *Papilio nireus* wings with no impurities demonstrated by XRD (Supplementary Fig. S2). The FESEM images reveal that the nano-hole arrays of BVO wing remain the quasi honeycomb architecture (Fig. 3b,d). Distinctions between the original wings and artificial ones mainly lie in the shrinkage (50~60%) of the dimensions. BVO wings derived from other butterfly wings are also obtained via this method (Supplementary Fig. S3).

**Figure 4:**
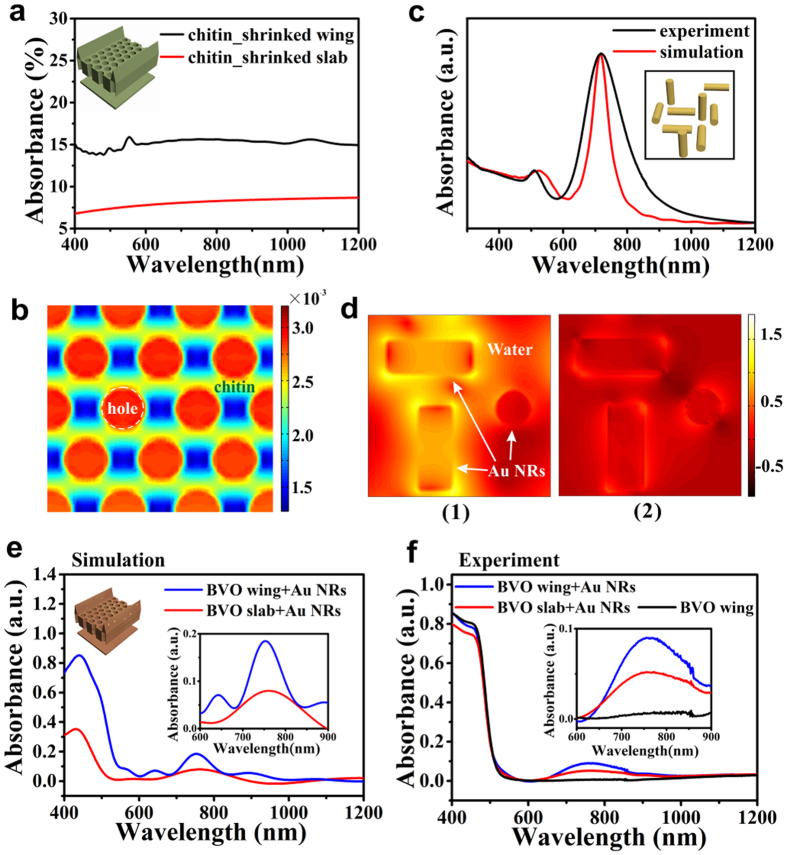
Simulative and experimental optical absorption properties of bio-inspired systems. (**a**) Simulative optical absorption and (**b**) a part of Poynting vector map (at 750 nm) at the cross-section of shrinked wing consisted of chitin. (**c**) Simulative and experimental optical absorption of Au NRs. (**d**) Log (R_E_) distribution of simulative Au NRs at (1) 716 nm, (2) 520 nm. (**e**) Simulative optical absorption of Au NRs-loaded BVO wing. (**f**) Experimental optical absorption of Au NRs-loaded BVO wing. Insets in (**a**,**c**), and upper side of (**e**) present the corresponding simulative models. Insets in lower side of (**e**,**f**) is the amplification from 600 nm to 900 nm in (**e**,**f**), respectively.

The absorption peak of Au NRs caused by LSPR can be tuned from ca. 600 nm to 1000 nm by changing their aspect ratio, so here Au NRs was selected as light harvesting nanoantennas. Au NRs were fabricated by a simple one-step method^44^ with high monodispersity (Fig. 3e) and yields (exceeding 90%). Figure 3f shows the average diameter and length of the Au NRs approximate 15 nm and 37.5 nm respectively, which corresponds to the aspect ratio of 2.5. The aspect ratio of Au NRs is controlled by the silver ion concentrations, resulting in the variation of absorption peaks^44^. Here, the LSPR peak was altered to 716 nm (Fig. 4c). To controlled assemble 1% Au NRs onto the surface of BVO wing (Fig. 1b) and the non-structured slab, a novel modified incipient wetness impregnation method was developed here (Fig. 3i). Direct impregnating BVO with colloidal Au NRs caused Au NRs’ reunion and inhomogeneous distribution on BVO surfaces (Supplementary Fig. S4). As the as-prepared CTAB-capped Au NRs are positively charged^45^, they could be tightly immobilized on the surface of BVO, which has been pretreated with EDTA/DMF suspension to gain abundant COO^−^ active sites (Fig. 3i). Figure 3g,h and Supplementary Fig. S5 prove that Au NRs well dispersed on BVO surfaces with arbitrary direction and no aggregation. It is also worth mentioning that the Au NRs colloidal solution changed from sepia color to transparency after the immersion of the BVO wing (Supplementary Fig. S6), in the meanwhile, the color of the transparent solution never changed even under vigorous shaking continuously, indicating practically all Au NRs were immobilized firmly onto the substrate.

### Simulative and Experimental Optical Absorption Properties of Bio-inspired Systems

FDTD method was further used to investigate the structural effects on light manipulation. Firstly, the fabricated structure with dimensions shrinkage by 50%–60% as described above was simulated. Since BVO has no response to far red-to-NIR light, the structural effects in NIR region can’t be highlighted using BVO as the constituent material (Supplementary Fig. S7). So chitin was hypothesized as the constituents in FDTD model to clarify the structural effects. The light absorption of shrinked wing reaches almost twice that of the slab from 400 nm to 1200 nm (Fig. 4a). This enhancement is much higher than that of the original wing (about 1.1 shown in Fig. 2a5), indicating the shrinked structures are even more favorable for visible-NIR light harvesting. To further indicate the working mechanism of the elaborate structure, especially the periodic hole arrays, the poynting vector map was studied. Distribution of poynting vector absolute value in the cross section of the structure at 750 nm was figured out from monitors of FDTD solutions (Fig. 4b). Obviously, color in the holes appears brighter than that of matrix, suggesting that a majority of light propagates through the holes. After being trapped into the holes, light is repeatedly reflected by hole walls, leading to the augment of effective path length for light absorption. Furthermore, the oblique ridges and the bottom DBR layer tend to guide light into the periodic hole arrays, where multiple reflections happens. Consequently, the 2D PC plays a vital role to assimilate NIR photons, while the other parts assist in regulating light into the 2D PC. The 3D elaborate quasi-honeycomb architecture, that does benefit NIR light harvesting, is supposed to be able to amplify the response of Au NRs to NIR light.

Experimental and simulative absorption spectrum of Au NRs were obtained by UV-VIS spectrophotometer and FDTD solutions respectively (Fig. 4c). The LSPR peak positions of experiment and simulation are both around 520 nm and 716 nm (Fig. 4c), which coincide well with each other. Figure 4d exhibits the distribution of Log (R_E_) in the cross section of Au NRs at 716 nm and 520 nm, corresponding to the longitudinal and transverse mode resonance^29^,^44^. R_E_ represents electric field intensity of the monitor plane divided by that of the incident light (R_E_ = |E|/|E_0_|). From Fig. 4d, we can work out that transverse resonance, which comes up regardless of Au NRs’ directions, is much inferior to longitudinal resonance that occurs only on the Au NRs direct along the polarization direction of light source^46^.

Afterwards, Au NRs-loaded BVO wing is simulated to confirm our speculation. After loading Au NRs onto BVO, an obvious absorption peak arises in the far-red to NIR region around 700~800 nm (Fig. 4e). The longitudinal resonance peak approximates 750 nm, revealing a red-shift compared to the Au NRs in water caused by medium dielectric constant change^46^. The absorption peak of Au NRs on BVO wing reaches almost twice that of non-structured counterparts, suggesting the remarkable enhancement of optical absorption caused by the butterfly wings’ 3D architectures. The experimental absorption spectrums of the as-prepared Au NRs-loaded BVO samples were also measured (Fig. 4f), consistent with the simulative results.

### Simulative LSPR-induced electric-field intensity in Bio-inspired Systems

**Figure 5:**
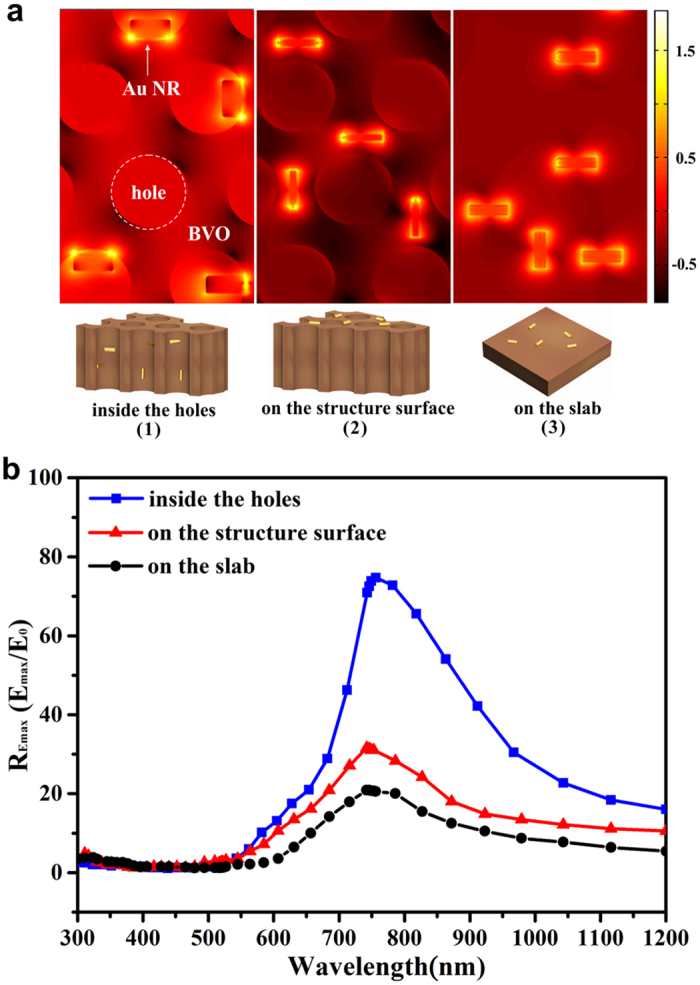
R_E_ in different architectures. (**a**) A part of Log (R_E_) distribution of simulative Au NRs-loaded BVO at 750 nm: (1) inside the holes of BVO wing, (2) at the surface of BVO wing, (3) at the surface of BVO slab. (**b**) R_Emax_ on different monitor planes of simulative Au NRs-loaded BVO models. Insets in (**a**) represent different Au NRs’ locations corresponding to the Log (R_E_) distributions above.

To explore the interactions between the structural effects and LSPRs of Au NRs, distribution of Log (R_E_) at 750 nm on specific planes, which represent three different configurations (Fig. 5a), is analyzed. Positions of the selected monitor planes are shown in Supplementary Fig. S8a. In the first configuration (Fig. 5a(1)), Au NRs are distributed inside the holes, which presents an intensive overlap between the 2D PC and Au NRs, while the second configuration (Fig. 5a(2)) presents a relatively weak overlap by monitoring Au NRs at the surface of the BVO wing. And the third configuration (Fig. 5a(3)) where Au NRs are attached to the upper surface of BVO slab is set as the non-structured model to eliminate structural effects. Maximal R_E_ of different configurations from 300 nm to 1200 nm is shown in Fig. 5b, revealing that the largest electric field intensity appears at around 750 nm where LSPR happens. Compared with Fig. 5a(3), the color of the region close to Au NRs in Fig. 5a(1) is brighter, indicating the electric field intensity near Au NRs in the holes of BVO wing (R_E_ reaches 75.89, blue line in Fig. 5b) is superior to that on the surface of BVO slab (R_E_ reaches 20.83, black line in Fig. 5b). Therefore, the electric field intensity is extremely strengthened by the architecture, and the structure-reinforced plasmon-photon coupling effect can be concluded (Fig. 1c). Afterwards, Fig. 5a(1,2) are taken into consideration. In Fig. 5a(1), the electric field intensity of air holes exceeds the matrix, so inside the holes fluctuates more light, most of which bounces back and forth by the hole walls, resulting in the increase of contacts between Au NRs and NIR light. Consequently, we can figure out that R_E_ of the region near the Au NRs inside holes surpasses that on the surface of the structure (Fig. 5a(2), R_E_ reaches 30.6, red line in Fig. 5b), suggesting that the periodic holes play a vital role in enhancing optical assimilation of Au NRs, and the ridges as well as the DBR act as assisting parts as depicted above. Moreover, the plasmon-photon coupling effect is dramatically amplified at the Au NRs/BVO interface (proved by Fig. 5a(1)), which will benefit photocatalytic performance by improving the rate of electron-hole formation^18^,^47^ (Fig. 1d).

### Photocatalytic Properties

To demonstrate the bio-inspired structural effects on far red-to-NIR solar energy utilization, we first present a practical and model reaction of photocatalytic gaseous isopropyl alcohol (IPA) degradation (Fig. 1e). The photo-oxidization of IPA to acetone is a one-electron process; after that, the acetone could be further oxidized into CO_2_ through a multi-electron oxidization process. Here, CO_2_ evolution rate was selected as the main comparison parameter while IPA degradation rate was also measured (Supplementary Fig. S9).

The photocatalytic conversions of IPA to CO_2_ over compared samples were carried out under far red-to-NIR irradiation (700~800 nm). There are two main mechanisms for plasmons-sensitized photocatalysis, namely charge injection mechanism and near-field electromagnetic enhancement mechanism^18^. Here the charge injection mechanism is not taken into account, because considering the fact that Au NRs are capped with a layer of CTAB, the contact of BiVO_4_ and Au NRs tends to be relatively poor, which causes a severe barrier for hot electrons transfer. So it is assumed that the charge injection from metal to photocatalytic semiconductor is hard to happen. As shown in Fig. 1d, photocatalysis of the system under far red-to-NIR irradiation can be explained by the near-field electromagnetic enhancement^33^,^34^, which is based on the interaction of BiVO_4_ with the strong surface plasmon resonance (SPR)-induced electric fields localized nearby at the Au NRs. In detail, Au NRs can be photo-excited by light with specific wavelength (700–800 nm in this study) due to SPR. Then the photo-excited plasmonic Au NRs are characterized by strong electric fields that are orders of magnitude higher than the field of photons used to photo-excite them. When BVO is brought into the proximity of a photo-excited plasmonic Au NRs, it encounters these intense fields. As the rate of electron-hole formation (excited electrons on conduction band and holes on valence band of BVO) is proportional to the local intensity of the electric field^47^,^48^, the electron-hole pairs can be formalized in some regions of the semiconductor because of the incredibly enhanced electric field. Therefore, the whole photocatalytic system can work under far red-to-NIR irradiation.

**Figure 6:**
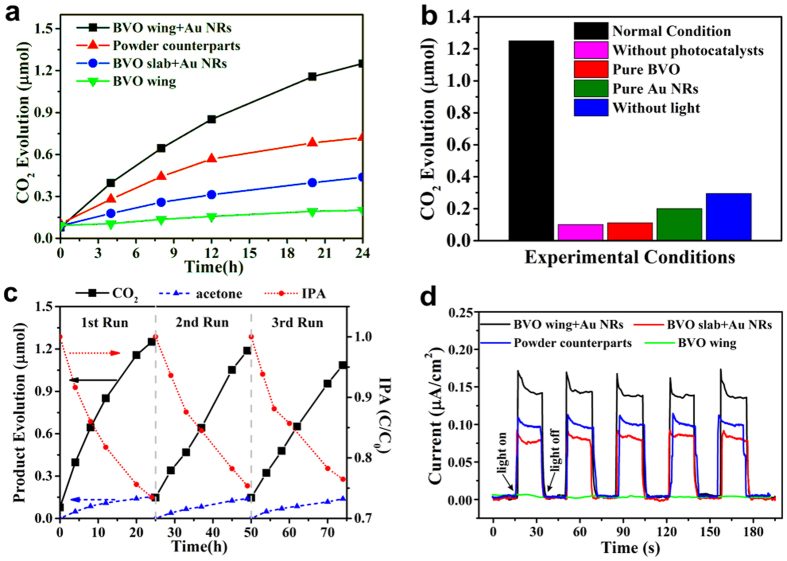
Photocatalytic activities. Photocatalytic IPA degradation (**a**) CO_2_ evolution as a function of illumination time on BVO series. (**b**) CO_2_ evolution after 24 h irradiation in reference experiments. (**c**) Cycling measurements of IPA degradation, acetone and CO_2_ generation. (**d**) Photocurrent response of BVO+Au NRs series under chopped monochromatic light at 750 nm.

Figure 6a indicates that 1 wt% Au NRs-loaded BVO wing (black line) evolved approximately 1.3 μmol of CO_2_ after 24 h irradiation (about 0.54 μmol h^−1^ g^−1^), which is 2.85 times as much as that of BVO slab + Au NRs (blue line). Furthermore, in order to emphasize the structural effects and exclude other factors, the as-prepared Au NRs-loaded BVO wing was thoroughly grinded to destroy the elaborated structure named as powder counterparts (red line). Obviously, there is a 42% decrease of photocatalytic performance after grinding, revealing the role of the architecture: (1) enhancing far red-to-NIR light harvesting as demonstrated by optical simulation (Fig. 4e) and experimental measurements (Fig. 4f) discussed above; (2) amplifying the plasmon-photon coupling effect of Au NRs. The underlying principle is interpreted by near-field electromagnetic mechanism^18^. As analyzed above, the sophisticated 3D structure helps amplify the electric-field intensity at the interface of BVO wing and Au NRs to more than 70 times that of the incident light (Fig. 5b), much larger than the non-structural one. Since the rate of electron-hole pair formation is proportional to the local intensity of electric field^18^,^47^,^48^, the photocatalytic performance is substantially reinforced as electron-hole formation is a dominant factor to photocatalysis.

Afterwards, in hopes of insights into the driving force of the photocatalytic property of Au NRs-loaded BVO composites, control experiments were conducted, while setting BVO wing + Au NRs under 700~800 nm illumination as the normal condition (Fig. 6b, black block). In Fig. 6b, pure BVO wing without Au NRs (Fig. 6b, red block) has negligible activity as pure BVO wings has no activity under far red-to-NIR irradiation, verifying the vital role of Au NRs in the process. The extremely small quantity of CO_2_ is brought in by inserting the syringe needle into the reactor during the process of extracting gas for ingredient analysis, which can be manifested by the fact that CO_2_ evolution in the condition “pure BVO without Au NRs” almost equals that in the condition “without photocatalysts (Fig. 6b, pink block)”. The sample “Pure Au NRs without BVO (Fig. 6b, green block)” shows little photocatalytic performance, which illustrates the significant effect of BVO (acts as host materials for photocatalysis), and the small amount of CO_2_ evolution is owing to photocatalysis/thermocatalysis of gold^49^ and the gas extraction process as mentioned above. Finally, a dark reaction over Au NRs-loaded BVO wing at 40 °C (the same temperature as the normal condition measured by a thermometer) was also implemented to eliminate the heating effect, only 0.29 μmol CO_2_ was detected after 24 h (Fig. 6b, blue block), much lower than the photocatalytic activity, suggesting the degradation of IPA was mainly caused by photocatalysis. The little performance is likely caused by the thermocatalysis of gold^49^,^50^,^51^ and the gas extraction process as mentioned above.

The durability of Au NRs-loaded BVO wing as a photocatalyst was checked. Figure 6c shows percentage of IPA, acetone and CO_2_ formation against illumination time during several cycles. After each run (24 h illumination), the sample was washed with deionized water followed by drying, and the reaction vessel was thoroughly purged with artificial air. The photocatalyst maintained its intrinsic activity during the three runs (Fig. 6c). No noticeable degradation was observed after more than 70 h of operation, indicating excellent durability. It is also worth mentioning that the photocatalytic system remain the same after three cycles (shown in Supplementary Fig. S10), indicating its stability during light irradiation.

Figure 6d shows the photocurrent-time functions of the as-prepared samples during repeating light on/light off cycles, which were obtained under 750 nm irradiation and zero applied bias voltage. Photocurrent of all samples are prompt and reproducible, while a controlled experiment using only BVO wing (green line) does not exhibit any photoresponse, indicating the vital role of Au NRs in the photocurrent under 750 nm irradiation. The transient decay observed in the light-on region is a result of surface recombination of electrons and holes^52^. In addition, we can easily figure out that the photocurrent of BVO wing + Au NRs (black line) obviously surpasses others, about 1.5 times and 2 times higher than that of powder counterparts (blue line) and BVO slab + Au NRs (red line) respectively, strongly verifying the structural effects mentioned above. Basically, the structure tends to enhance optical absorption and magnify the LSPR-induced electric field, promoting electron-hole separation, thus improving the photocurrent generation.

## Discussion

To summarize, we have demonstrated a new bio-inspired platform governed by biological elaborated architectures for novel efficient NIR responsive photocatalysis. Structural improvements were verified by the fact that photocatalytic degradation and photoelectrochemical performance of materials with the structure inherited by butterfly wings approximated 2.85 times and 2 times than the non-structural ones. Spectroscopic characterization and FDTD simulations were performed to obtain further insight into the mechanisms for structural effects: (1) Enhancing far red-to-NIR light harvesting ability. The nano-pores in the structure where multi-reflection occurs play a vital role, while other parts help light propagate into the pores. The reported enhancement effect is generic that it can be applied to a wide range of biological light harvesting architectures. (2) Enhancing the electric field (E-field) amplitude of LSPs due to the coupling of the LSPs with the 3D architectures of the butterfly wings, especially when the Au NRs are placed inside the pores of the architectures. Therefore, the photocatalytic performance was considerably improved due to the largely increased rate of electron-hole pair formation, which is proportional to the local intensity of electric field. The study demonstrates that butterfly wings scales, as a concept prototype would pave a new pathway for morphology control, which hints at a more general principle for NIR photocatalysis with a large variety of optimized biological geometries and the underlying mechanisms. These nanoarchitectures of biological systems suggest opportunities for future nanoengineered structures in providing improvements in NIR response. Furthermore, this is also a pioneering work on the light-matter interaction between plasmonic metal nanostructures and semiconductors with unique 3D micro/nanoarchitectures, which could provide useful information for the optimized loading of plasmonic antennas. The observation and in-depth understanding of such light-matter interaction is the key to the development of plasmonic metal nanostructures based devices. Additionally, here gold NRs serve as far red-to-NIR light-harvesting antennas, absorption of wavelength can be tuned to cover full solar spectrum or extend to longer wavelength (e.g. V-Shaped Au NRs) with other plasmonic nanostructures. Thus, in principle, it is possible to design a bio-inspired system that may harvest photons over the entire solar spectrum. While here we choose BVO as our target material in this proof-of-concept study, the strategy described herein could be generally applied using many other photocatalysts. We view this study to be the first step in the development of a new methodology for NIR photocatalysis *via* morphology control. Next steps will be to engineer the NIR responsive elaborated structures with artificial nanofabrication methods such as electron beam lithography, photolithography, 3D printing technology, self-assembly and so forth. We anticipate that this bio-inspired strategy could be extended to other solar conversion systems such as solar cells, thermoelectric devices, NIR photodetectors and so forth.

## Methods

### Optical simulation

Optical simulations was conducted by FDTD solutions using the finite-difference time-domain (FDTD) method. The simulation model consisted of FDTD region (boundary conditions: PML in z direction, Periodic in x and y direction, mesh setting: auto non-uniform, mesh accuracy: 3), light source (plane wave, polarizes along x and y direction, wavelength: 300–1200 nm), monitors (Frequency-domain field and power), mesh (encompassed Au NRs, dx = dy =dz = 1 nm), simulation model. Schematic diagram of the simulation model is shown in Supplementary Fig. S8a. Structures and dimensions of the simulation systems were taken from SEM images. The slab model was established by pressing the corresponding structural model into a rectangle slab with equal length, width and volume (Supplementary Fig. S8b). Original wing scales are composed of chitin and their respective pigments. In order to reveal the effects of architectures, the refractive index (RI) of the ingredient in our models is assumed to be 1.56 + 0.06i regardless of the wavelength^26^. Dielectric constant of BiVO_4_ and Au (gold) as the input parameters are from reported data^53^ and FDTD solutions (Johnson and Christy), respectively. Absorbance of the simulation system was assumed to be equal to 1-transmissivity-reflectivity. Transmissivity, reflectivity and power flux density were obtained from different Frequency-domain field and power monitors.

### Synthesis of bismuth vanadate systems

Bismuth vanadate with quasi honeycomb structures was synthesized by a modified sol-gel method. In a typical procedure, firstly, all the ventral scales and the black parts of *Papilio nireus* butterfly wings were removed, leaving the residual green parts for the synthesis. The wing scales were immersed in a mixed solution that consisted of acetic acid and ethanol (1:1 in volume) for 12 h at 30 °C, then rinsed with ethanol twice before drying in air. Afterwards, a BVO precursor was prepared as follows 30 °C: (1) 19.4 g Bi(NO_3_)_3_·5H_2_O was dissolved in a mixture containing 60 mL glycerol and 80 mL ethanol at 70 °C under magnetic stirring; (2) 4.68 g NH_4_VO_3_ was dissolved in 20 mL TMAH under gently shaking; (3) these two solutions were mixed together at 70 °C (large amounts of yellow deposit appeared) followed by adding concentrated nitric acid (65%) promptly until the solution became transparent. The pretreated butterfly scales were carefully placed into the precursor and kept at room temperature overnight. Next, the butterfly scales were pulled out, rinsed with ethanol and air dried. In the last stage, the samples were sintered under continuous air flowing at 500 °C for 6 h to remove the organic constituents. BVO without hierarchical structures (BVO slab) was prepared with the same steps described above, except applying the substrates of butterfly wings scales. Substrates of butterfly wings were obtained by using alcohol swabs to scrape the scales off the wings.

### Synthesis of Au nanorods

Highly monodispersed Au nanorods with LSPR peaks at 716 nm were prepared by a simple one-step method^44^. Firstly, several chemicals were sequentially added into 7.125 mL of CTAB (0.11 M) solution under slight shaking followed by standing for 5 min. The final concentrations of these chemicals were [CTAB] = 0.1 M, [AgNO_3_] = 0.086 mM, [HAuCl_4_] = 0.4 mM, [hydroquinone] = 5.26 mM. The volume was 7.6 mL. Then, 0.76 μL of NaBH_4_ dissolved in ice water (17 mM) was added under vigorous stirring and kept standing for 12 h. All experiments were conducted at 30 °C. Finally, the Au nanorods colloidal solution were centrifuged at 8000 rpm twice and redispersed in deionized water to remove redundant CTAB.

### Loading of 1 wt% Au NRs onto BVO photocatalysts

Au NRs-loaded BVO with longitudinal plasmon resonance peak at 750 nm was synthesized *via* impregnating colloidal gold nanorods with BVO. Specifically, a pretreating suspension was prepared by blending Ethylene Diamine Tetraacetic Acid (EDTA) with Dimethyl Formamide (DMF) according to the mass ratio of 1:10. Then 5 mL supernatant of the suspension was sucked up and injected into the culture dish (60 mm radius) overspread with 0.1 g BVO samples drop by drop, followed by heat preservation at 110 °C for 6 h. After that, the samples were rinsed with hot deionized water three times, impregnated with 1.6 mL 0.65 mg/mL Au nanorods colloidal solution, kept in an incubator encapsulated with tinfoil at 60 °C for 6 h. Finally, they were air dried at 60 °C overnight.

### Characterization

Microstructures of the samples were observed by a scanning electron microscope (SEM, Hitachi S-4800) and a transmission electron microscope (TEM, JEM-2100F). XRD measurements were performed using a X-ray diffractometer (Rigaku, D-max/2550) with Cu Kα radiation (λ = 0.154 nm). The optical absorption measurements were collected on a UV-Vis-NIR spectrophotometer (PerkinElmer, 750S). For the sake of highlighting structural effects on light absorption, we dropped bromoform on the butterfly wings before measuring the optical absorption, then compared their absorption spectrum with that of butterfly wings in air^39^. The structural effects are eliminated because bromoform has the similar reflective index as chitin that constitutes butterfly scales. For the sake of comparing absorption spectrums of the as-prepared BVO samples, 0.02 g of different samples occupied the same area of 4 cm^2^ on the BiSO_4_ sample stage was adopted.

### Photocatalytic measurements

Photocatalytic gaseous IPA degradation was carried out in a system composed of a light source (300 W xenon lamp) and a 600 mL sealed quartz reactor. Optical cut-off filters (Beijing perfectlight technology co., LTD) were applied to restrict the wavelength of incident light to 700~800 nm. To meaure the photocatalytic performance, IPA, acetone and CO_2_ were measured by a gas chromatography (SP6900, Beijing Aulight technology co., LTD) equipped with a flame ionization detector (FID). For each reaction, 100 mg sample was evenly spread over a dish with a diameter of 60 mM in the reactor. After the sample was sealed in the vessel, the inside atmosphere of the reactor was exchanged by artificial air [V(N_2_) : V(O_2_) = 9 : 1] for 10 min to remove gaseous impurities. Then gaseous IPA was injected into the vessel (the initial concentration of IPA was about 200 ppm). The sample was kept in the dark for 1 h to ensure an adsorption-desorption equilibrium of IPA on the sample before irradiation. When the reaction began, at set intervals, 1 mL gas was extracted from the reactor by a 1 mL syringe with gas tight brazed ends (1 mL, Shanghai Gaoge industrial and trading co., LTD), followed by injecting to the gas chromatography to measure the content of each gas (IPA, acetone, CO_2_).

### Photoelectrochemical activity measurement

Photocurrent was measured in a two-electrode system with an electrochemical workstation. The two-electrode system (without a reference electrode, under zero applied bias voltage) in a quartz container was composed of a Pt-sheet counter electrode and a working electrode. A 0.5 M Na_2_SO_4_ aqueous solution was used as the electrolyte solution. To fabricate the working electrode, 3 mg samples were spreading uniformly on a 1 cm^2^ ITO glass before dropping 20 μL of 0.5 mM Nafion ethanol solution on the samples. Then the ITO glass decorated with samples were dried at 60 °C in air for 2 h. The light source was consisted of a xenon lamp (7ILX500, SOFN Instruments Co. Ltd.) and a double grating monochromator (7ISW15, SOFN Instruments Co. Ltd.), providing monochromatic light at 750 nm.

## Additional Information

**How to cite this article**: Yan, R. *et al*. Bio-inspired Plasmonic Nanoarchitectured Hybrid System Towards Enhanced Far Red-to-Near Infrared Solar Photocatalysis. *Sci. Rep.* **6**, 20001; doi: 10.1038/srep20001 (2016).

## Supplementary Material

Supplementary Information

## References

[b1] Osterloh F. E. Inorganic nanostructures for photoelectrochemical and photocatalytic water splitting. Chem. Soc. Rev. 42, 2294–2320 (2013). 23072874 10.1039/c2cs35266d

[b2] Chirilă A. . Potassium-induced surface modification of Cu (In, Ga) Se_2_ thin films for high-efficiency solar cells. Nat. Mater. 12, 1107–1111 (2013). 24185758 10.1038/nmat3789

[b3] Tong H. . Nano-photocatalytic Materials: Possibilities and Challenges. Adv. Mater. 24, 229–251 (2012). 21972044 10.1002/adma.201102752

[b4] Yang S. B. . Exfoliated Graphitic Carbon Nitride Nanosheets as Efficient Catalysts for Hydrogen Evolution Under Visible Light. Adv. Mater. 25, 2452–2456 (2013). 23450777 10.1002/adma.201204453

[b5] Zhou H. . Artificial Inorganic Leafs for Efficient Photochemical Hydrogen Production Inspired by Natural Photosynthesis. Adv. Mater. 22, 951–956 (2010). 20217818 10.1002/adma.200902039

[b6] Zhou H. . Leaf-architectured 3D Hierarchical Artificial photosynthetic system of perovskite titanates towards CO_2_ Photoreduction into hydrocarbon fuels. Sci. Rep. 3, 1667 (2013). 10.1038/srep01667 PMC3627189 23588925

[b7] Wang G. . Cu_2_(OH)PO_4_, a Near-Infrared-Activated Photocatalyst. Angew. Chem. Int. Ed. 52, 4810–4813 (2013). 10.1002/anie.201301306 23554138

[b8] Sang Y. H. . From UV to Near-Infrared, WS_2_ Nanosheet: A novel photocatalyst for full solar light spectrum photodegradation. Adv. Mater. 27, 363–369 (2015). 25413166 10.1002/adma.201403264

[b9] Zhang Z. J. & Wang W. Z. Infrared-light-induced photocatalysis on BiErWO_6_. Dalton. Trans. 42, 12072–12074 (2013). 23629148 10.1039/c3dt50470k

[b10] Tian J. . A Bi_2_WO_6_-Based Hybrid photocatalyst with broad spectrum photocatalytic properties under UV, Visible, and Near-Infrared Irradiation. Adv. Mater. 25, 5075–5080 (2013). 23852936 10.1002/adma.201302014

[b11] Xi G. . Ultrathin W_18_O_49_ Nanowires with Diameters below 1 nm: Synthesis, Near‐Infrared Absorption, Photoluminescence, and Photochemical Reduction of Carbon Dioxide. Angew. Chem. Int. Ed. 51, 2395–2399 (2012). 10.1002/anie.201107681 22282345

[b12] Chen X. B., Liu L., Yu P. Y. & Mao S. S. Increasing solar absorption for photocatalysis with black hydrogenated titanium dioxide Nanocrystals. Science 331, 746–750 (2011). 21252313 10.1126/science.1200448

[b13] Obregon S. & Colon G. Evidence of upconversion luminescence contribution to the improved photoactivity of erbium doped TiO_2_ systems. Chem. Commun. 48, 7865–7867 (2012). 10.1039/c2cc33391k 22785495

[b14] Li H. T. . Near-infrared light controlled photocatalytic activity of carbon quantum dots for highly selective oxidation reaction. Nanoscale 5, 3289–3297 (2013). 23467384 10.1039/c3nr00092c

[b15] Pak C. . Extending the limit of low-energy photocatalysis: Dye reduction with PbSe/CdSe/CdS Core/Shell/Shell Nanocrystals of Varying Morphologies under Infrared Irradiation. J. Phys. Chem. C 116, 25407–25414 (2012).

[b16] Wang H. L. . Semiconductor heterojunction photocatalysts: design, construction, and photocatalytic performances. Chem. Soc. Rev. 43, 5234–5244 (2014). 24841176 10.1039/c4cs00126e

[b17] Liu L. Q., Ouyang S. X. & Ye J. H. Gold-Nanorod-Photosensitized Titanium Dioxide with Wide-Range Visible-Light Harvesting Based on Localized Surface Plasmon Resonance. Angew. Chem. Int. Ed. 52, 6689–6693 (2013). 10.1002/anie.201300239 23666880

[b18] Linic S., Christopher P. & Ingram D. B. Plasmonic-metal nanostructures for efficient conversion of solar to chemical energy. Nat. Mater. 10, 911–921 (2011). 22109608 10.1038/nmat3151

[b19] Antonietti M. & Fratzl P. Biomimetic Principles in Polymer and Material Science. Macromol. Chem. Phys. 211, 166–170 (2010).

[b20] Wegst U. G. K., Bai H., Saiz E., Tomsia A. P. & Ritchie R. O. Bioinspired structural materials. Nat. Mater. 14, 23–36 (2015). 25344782 10.1038/nmat4089

[b21] Sun J. H. . Bioinspired hollow semiconductor nanospheres as photosynthetic nanoparticles. Nat. Commun. 3, (2012).

[b22] Liu J. & Antonietti M. Bio-inspired NADH regeneration by carbon nitride photocatalysis using diatom templates. Energy Environ. Sci. 6, 1486–1493 (2013).

[b23] Behrendt L. . Biofilm Growth and Near-Infrared Radiation-Driven Photosynthesis of the Chlorophyll d-Containing Cyanobacterium Acaryochloris marina. Appl. Environ. Microb. 78, 3896–3904 (2012). 10.1128/AEM.00397-12 PMC3346409 22467501

[b24] Spinner M., Kovalev A., Gorb S. N. & Westhoff G. Snake velvet black: Hierarchical micro- and nanostructure enhances dark colouration in Bitis rhinoceros. Sci. Rep. 3, 1846 (2013). 10.1038/srep01846 PMC3655483 23677278

[b25] Campbell A. L., Bunning T. J., Stone M. O., Church D. & Grace M. S. Surface ultrastructure of pit organ, spectacle, and non pit organ epidermis of infrared imaging bold snakes: a scanning probe and scanning electron microscopy study. J. Struct. Biol. 126, 105–120 (1999). 10388622 10.1006/jsbi.1999.4121

[b26] Van Hooijdonk E., Vandenbem C., Berthier S. & Vigneron J. P. Bi-functional photonic structure in the Papilio nireus (Papilionidae): modeling by scattering-matrix optical simulations. Opt. Express 20, 22001–22011 (2012). 23037350 10.1364/OE.20.022001

[b27] Yu K. L., Fan T. X., Lou S. & Zhang D. Biomimetic optical materials: Integration of nature’s design for manipulation of light. Prog. Mater. Sci. 58, 825–873 (2013).

[b28] Pris A. D. . Towards high-speed imaging of infrared photons with bio-inspired nanoarchitectures. Nat. Photonics 6, 195–200 (2012).

[b29] Chen H. J., Shao L., Li Q. & Wang J. F. Gold nanorods and their plasmonic properties. Chem. Soc. Rev. 42, 2679–2724 (2013). 23128995 10.1039/c2cs35367a

[b30] Xia Y. N., Xiong Y. J., Lim B. & Skrabalak S. E. Shape-Controlled Synthesis of Metal Nanocrystals: Simple Chemistry Meets Complex Physics? Angew. Chem. Int. Ed. 48, 60–103 (2009). 10.1002/anie.200802248 PMC2791829 19053095

[b31] Mubeen S. . An autonomous photosynthetic device in which all charge carriers derive from surface plasmons. Nat. Nanotech. 8, 247–251 (2013). 10.1038/nnano.2013.18 23435280

[b32] Silva C. G., Juarez R., Marino T., Molinari R. & Garcia H. Influence of Excitation Wavelength (UV or Visible Light) on the Photocatalytic Activity of Titania Containing Gold Nanoparticles for the Generation of Hydrogen or Oxygen from Water. J. Am. Chem. Soc. 133, 595–602 (2011). 21142160 10.1021/ja1086358

[b33] Cushing S. K. . Photocatalytic Activity Enhanced by Plasmonic Resonant Energy Transfer from Metal to Semiconductor. J. Am. Chem. Soc. 134, 15033–15041 (2012). 22891916 10.1021/ja305603t

[b34] Seh Z. W. . Janus Au-TiO_2_ Photocatalysts with Strong Localization of Plasmonic Near-Fields for Efficient Visible-Light Hydrogen Generation. Adv. Mater. 24, 2310–2314 (2012). 22467121 10.1002/adma.201104241

[b35] Chen H. M. . Plasmon Inducing Effects for Enhanced Photoelectrochemical Water Splitting: X-ray Absorption Approach to Electronic Structures. Acs Nano 6, 7362–7372 (2012). 22849358 10.1021/nn3024877

[b36] Kinoshita S. & Yoshioka S. Structural colors in nature: The role of regularity and irregularity in the structure. Chemphyschem 6, 1442–1459 (2005). 16015669 10.1002/cphc.200500007

[b37] Bae W. G. . 25th Anniversary Article: Scalable Multiscale Patterned Structures Inspired by Nature: the Role of Hierarchy. Adv. Mater. 26, 675–699 (2014). 24353032 10.1002/adma.201303412

[b38] Sato O., Kubo S. & Gu Z. Z. Structural Color Films with Lotus Effects, Superhydrophilicity, and Tunable Stop-Bands. Accounts. Chem. Res. 42, 1–10 (2009). 10.1021/ar700197v 18837520

[b39] Yu K. L. . Structural integration design for enhanced photoluminescence in butterfly scales. Soft Matter 9, 2614–2620 (2013).

[b40] Zhao Q. B. . Art of blackness in butterfly wings as natural solar collector. Soft Matter 7, 11433–11439 (2011).

[b41] Ono Y., Kimura Y., Ohta Y. & Nishida N. Antireflection effect in ultrahigh spatial-frequency holographic relief gratings. Appl. Optics 26, 1142–1146 (1987). 10.1364/AO.26.001142 20454283

[b42] Abdi F. F. . Efficient solar water splitting by enhanced charge separation in a bismuth vanadate-silicon tandem photoelectrode. Nat. Commun. 4, 2195 (2013). 23893238 10.1038/ncomms3195

[b43] Wang J. R. & Osterloh F. E. Limiting factors for photochemical charge separation in BiVO_4_/Co_3_O_4_, a highly active photocatalyst for water oxidation in sunlight. J. Meter. Chem. A 2, 9405–9411 (2014).

[b44] Zhang L. M. . Efficient and Facile Synthesis of Gold Nanorods with Finely Tunable Plasmonic Peaks from Visible to Near-IR Range. Chem. Mater. 26, 1794–1798 (2014).

[b45] Guo L. H., Zhou X. D. & Kim D. H. Facile fabrication of distance-tunable Au-nanorod chips for single-nanoparticle plasmonic biosensors. Biosens. Bioelectron. 26, 2246–2251 (2011). 21035320 10.1016/j.bios.2010.09.042

[b46] Link S., Mohamed M. B. & El-Sayed M. A. Simulation of the optical absorption spectra of gold nanorods as a function of their aspect ratio and the effect of the medium dielectric constant. J. Phys. Chem. B 103, 3073–3077 (1999).

[b47] Lee J. . Bioconjugated Ag nanoparticles and CdTe nanowires: Metamaterials with field-enhanced light absorption. Angew. Chem. Int. Ed. 45, 4819–4823 (2006). 10.1002/anie.200600356 16802399

[b48] Anger P., Bharadwaj P. & Novotny L. Enhancement and quenching of single-molecule fluorescence. Phys. Rev. Lett. 96, (2006). 10.1103/PhysRevLett.96.113002 16605818

[b49] Linic S., Aslam U., Boerigter C. & Morabito M. Photochemical transformations on plasmonic metal nanoparticles. Nat. Mater. 14, 567–576 (2015). 25990912 10.1038/nmat4281

[b50] Kowalska E., Mahaney O. O. P., Abe R. & Ohtani B. Visible-light-induced photocatalysis through surface plasmon excitation of gold on titania surfaces. Phys. Chem. Chem. Phys. 12, 2344–2355 (2010). 20449347 10.1039/b917399d

[b51] Liu L. Q. . Plasmonic Janus-Composite Photocatalyst Comprising Au and C-TiO2 for Enhanced Aerobic Oxidation over a Broad Visible-Light Range. Adv. Funct. Mater. 24, 7754–7762 (2014).

[b52] Peter L. M. Dynamic aspects of semiconductor photoelectrochemistry. Chem. Rev. 90, 753–769 (1990).

[b53] Zhao Z. Y., Li Z. S. & Zou Z. G. Electronic structure and optical properties of monoclinic clinobisvanite BiVO_4_. Phys. Chem. Chem. Phys. 13, 4746–4753 (2011). 21283853 10.1039/c0cp01871f

