# Supplementary material for: Bio-inspired Plasmonic Nanoarchitectured Hybrid System Towards Enhanced Far Red-to-Near Infrared Solar Photocatalysis
Source: Sci Rep. 2016 Jan 28;6:20001. doi: 10.1038/srep20001 (PMC4730232; doi:10.1038/srep20001)
Supplement: Supplementary Information [file srep20001-s1.doc]

Supplementary Information

Bio-inspired Plasmonic Nanoarchitectured Hybrid System Towards Enhanced Far Red-to-Near Infrared Solar Photocatalysis

*Runyu Yan^1^, Min Chen^1^, Han Zhou*^1^, Tian Liu^1^, Xingwei Tang^1^, Ke Zhang^1^, Hanxing Zhu^4^, Jinhua Ye^2,3^, Di Zhang^1^ and Tongxiang Fan*^1^*

^1^State Key Lab of Matel Matrix Composites, Department of Materials Science and Engineering, Shanghai Jiaotong University, Shanghai, 200240, China

Corresponding authors Email: hanzhou_81@sjtu.edu.cn, txfan@sjtu.edu.cn

^2^International Center for Materials Nanoarchitectonics (WPI-MANA) and Environmental Remediation Materials Unit, National Institute for Materials Science (NIMS), 1-1, Namiki, Tsukuba, Ibaraki 305-0044, Japan

^3^TU−NIMS Joint Research Center, School of Materials Science and Engineering, Tianjin University, 92 Weijin Road, Nankai District, Tianjin 300072, P. R. China

^4^School of Engineering, Cardiff University, Cardiff, CF24 3AA, UK

**Contents:**

**Supplementary Figure S1.** Experimental optical absorption vs wavelength curves of butterfly wings.

**Supplementary Figure S2.** XRD pattern of BVO wing.

**Supplementary Figure S3.** Morphology of original and artificial butterfly wings with other structures.

**Supplementary Figure S4.** FESEM images of Au NRs-loaded BVO by direct incipient wetness impregnation method.

**Supplementary Figure S5.** TEM images of Au NRs-loaded BVO wing.

**Supplementary Figure S6.** Appearance of the as-prepared BVO wing + Au NRs sample during the process of loading Au NRs.

**Supplementary Figure S7.** Simulative optical absorption curve of BVO with/without shrinked structure.

**Supplementary Figure S8.** Schematic diagram of optical simulation using FDTD solutions and construction of the slab model.

**Supplementary Figure S9.** Photocatalytic IPA degradation activities measured by IPA.

**Supplementary Figure S10.** SEM and TEM images of Au NRs-loaded BVO wing after three cycles of photocatalytic measurement.

**Supplementary Table S1.** Dimension figure of different structures.

**
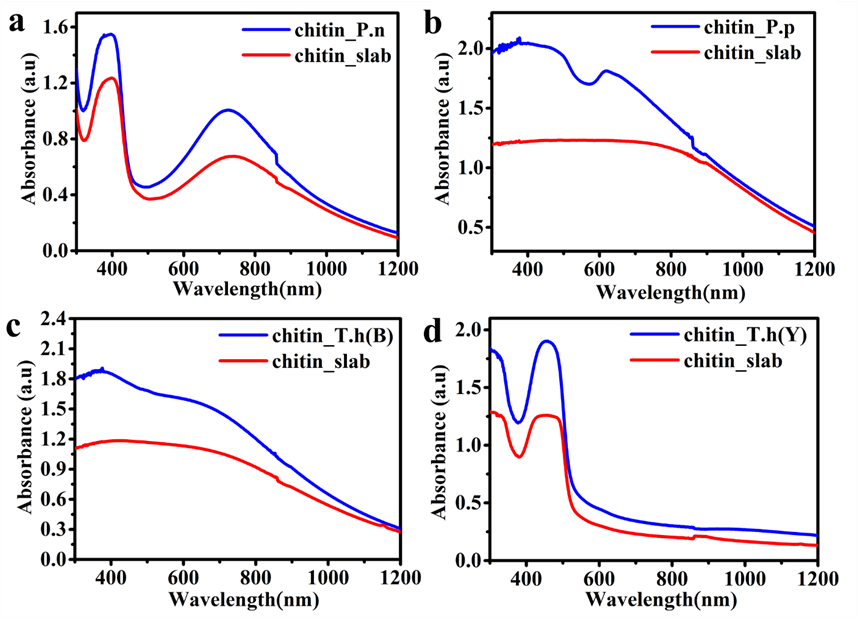
**

**Supplementary Figure S1.** **Experimental optical absorption *vs* wavelength curves of butterfly wings**. (**a**) *Papilio nireus,* (**b**) *Papilio paris,* (**c**) *Troides helena*’s forewing and (**d**) *Troides helena*’s underwing. Optical absorption at 700 nm - 800 nm enhanced by the four kinds of architectures is verified. However, experimental results obviously differ from the simulative ones (Figure 1a5-d5) in curve shapes, which is ascribed to the distinction of constituent materials between experiment and simulation. In detail, pigments distributed over real scales play an important role in regulating light (causing absorption peaks), while pigments are neglected in simulative scales to simplify the structure models (the structural effects can also be highlighted without pigments). Moreover, the optical parameters of chitin in simulation are more or less inconsistent with that of the genuine chitin. Especially at relatively long wavelength, genuine chitin absorbs light so scarcely that the absorption curves of structural scales reach closely to that of non-structural ones, but in contrast, the simulative chitin is able to interact with long wavelength light, so that the structural effects can be emphasized.

**
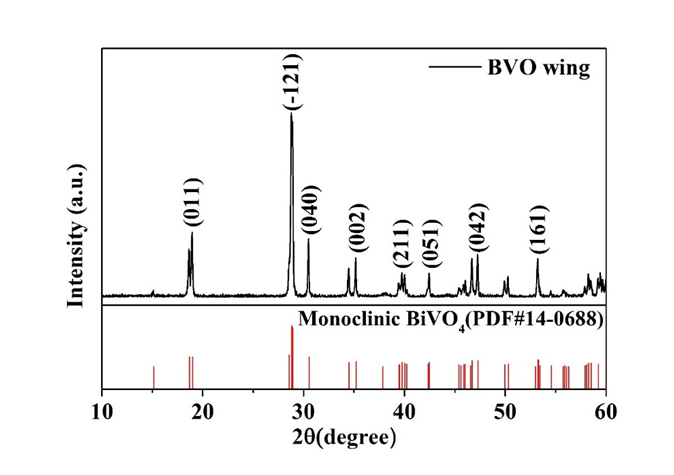
**

**Supplementary Figure S2. XRD pattern of BVO wing.**

**
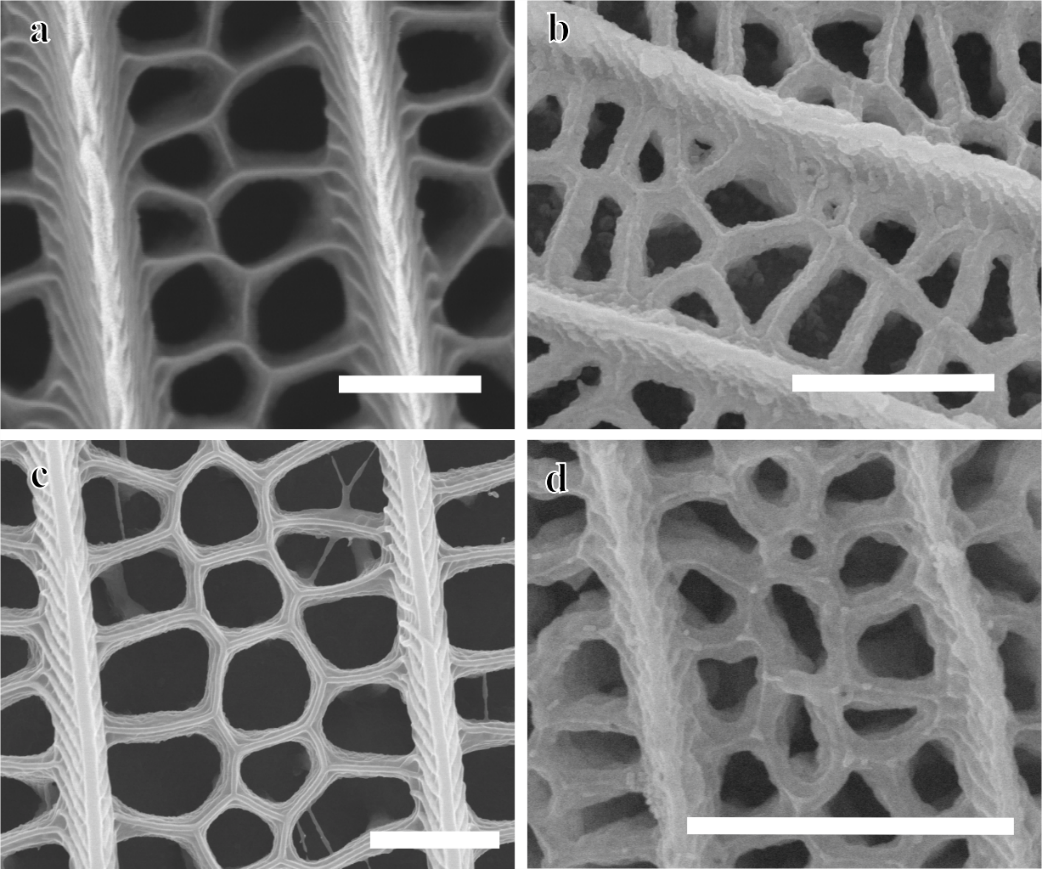
**

**Supplementary Figure S3. Morphology of original and artificial butterfly wings with other structures.** FESEM images of (**a**) original Troides helena’s forewing, (**b**) artificial BVO Troides helena’s forewing, (**c**) original Papilio paris wing and (**d**) artificial BVO Papilio paris wing. All scale bars equal 1 µm.


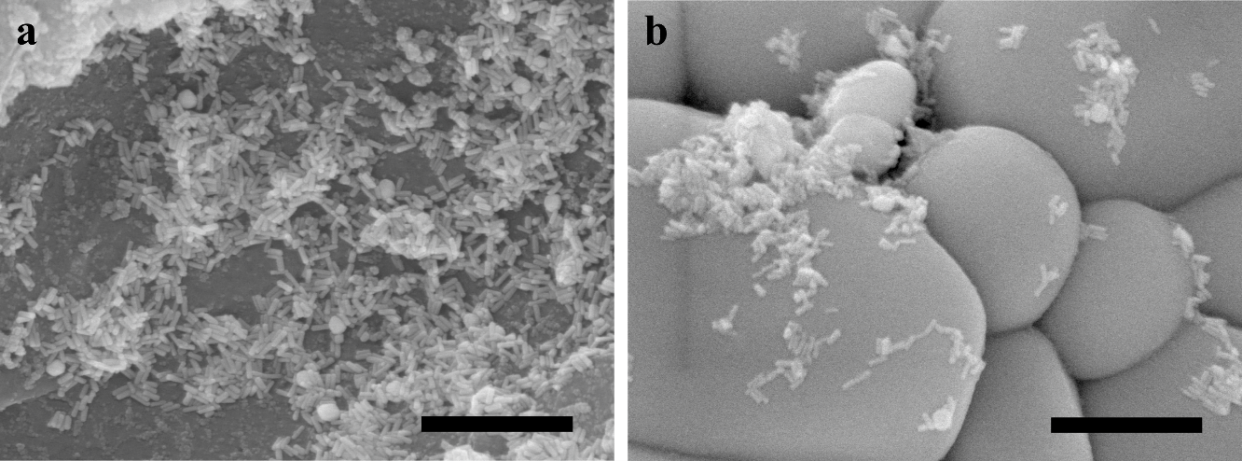


**Supplementary Figure S4. FESEM images of Au NRs-loaded BVO by direct incipient wetness impregnation method.** Both images show the Au NRs’ reunion and inhomogeneous distribution on the surface of BVO. Scale bars equals 1 µm.


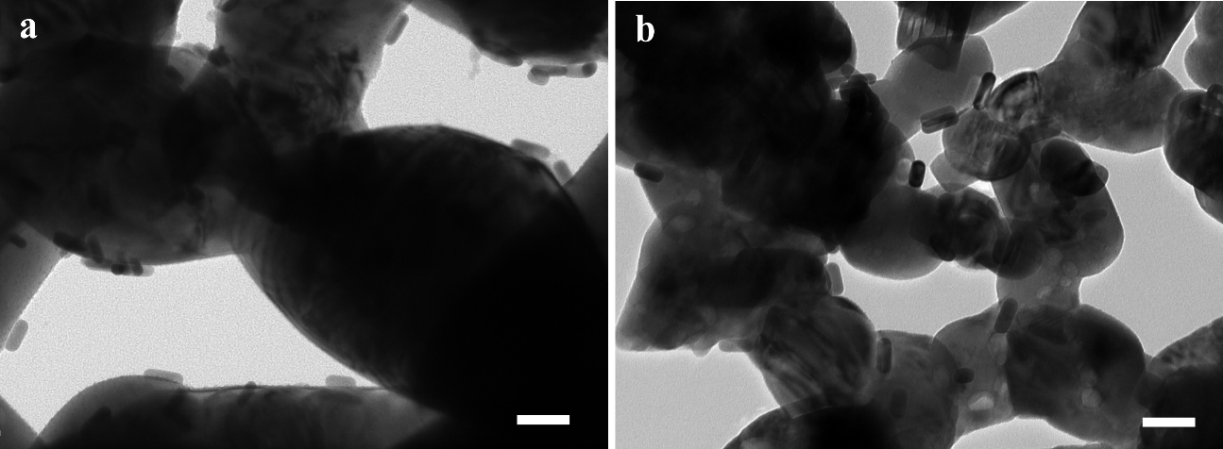


**Supplementary Figure S5. TEM figures of Au NRs-loaded BVO wing.** Uniform distribution of Au NRs was proved. Scale bar equals 50 nm.

**
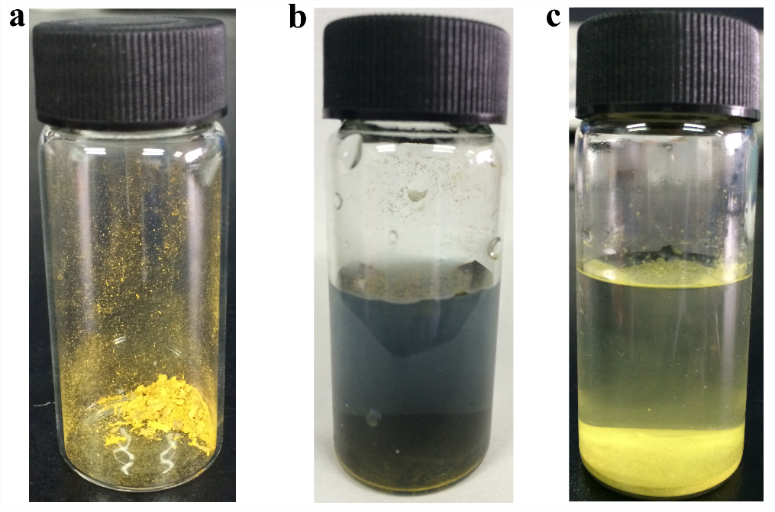
**

**Supplementary Figure S6. Appearance of the as-prepared BVO wing + Au NRs sample during the process of loading Au NRs.** (**a**) Pretreated BVO wing; (**b**) BVO wing immersed in the solution of Au NRs; (**c**) after 4 hours’ immersion at 60 °C.

**
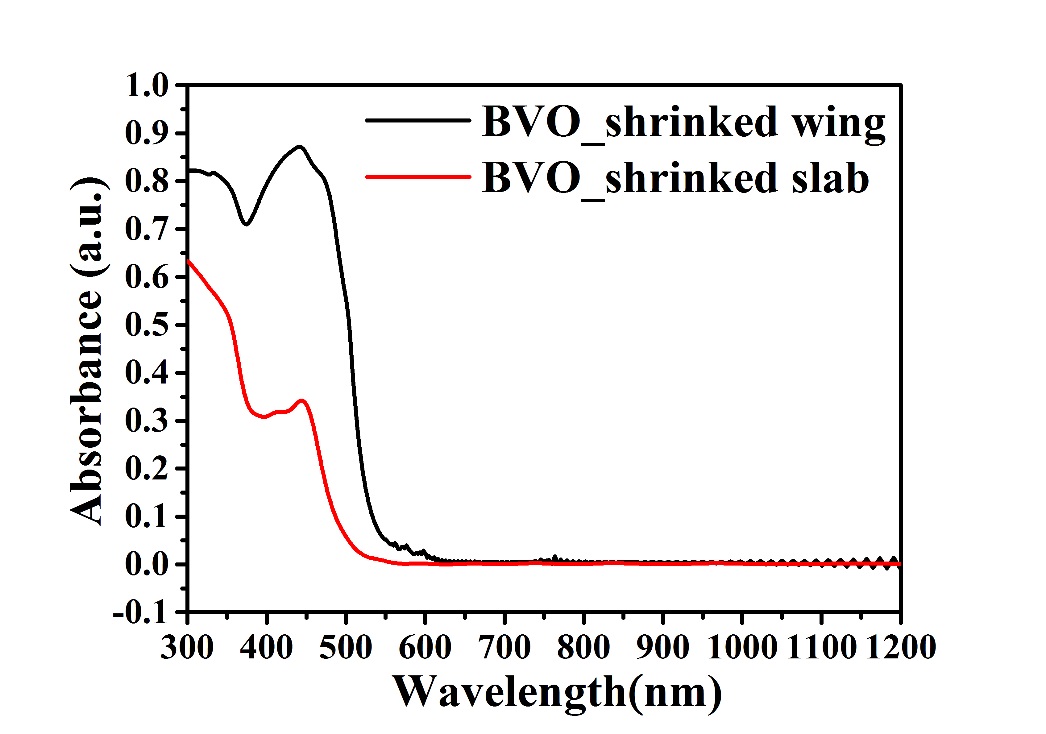
**

**Supplementary Figure S7. Simulative optical absorption curve of BVO with/without** **shrinked structure.**

**
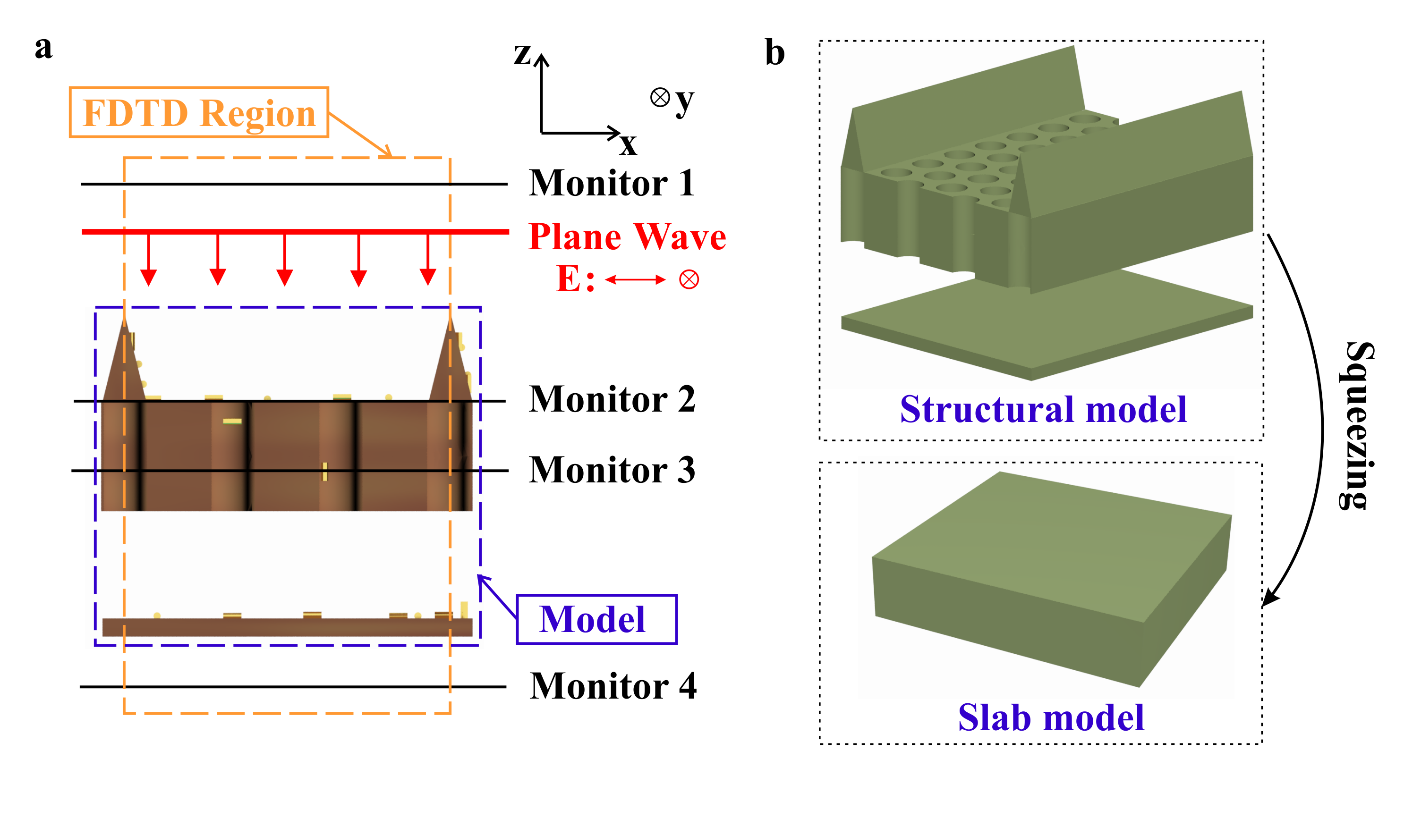
**

**Supplementary Figure S8. Schematic diagram of optical simulation using FDTD solutions and construction of the slab model.** (**a**) Schematic diagram of optical simulation using FDTD solutions. Different simulative samples were conducted by replacing the model in this figure. Monitor 1 and 4 were employed for reflectivity and transmissivity calculation respectively, while Monitor 2 and 3 that represented the surface and the cross-section of BVO wing were set for calculating field intensity distribution. (**b**) Schematic diagram of construction of the slab model. The slab model was established by squeezing the corresponding structural model into a rectangle slab with equal length, width and volume.


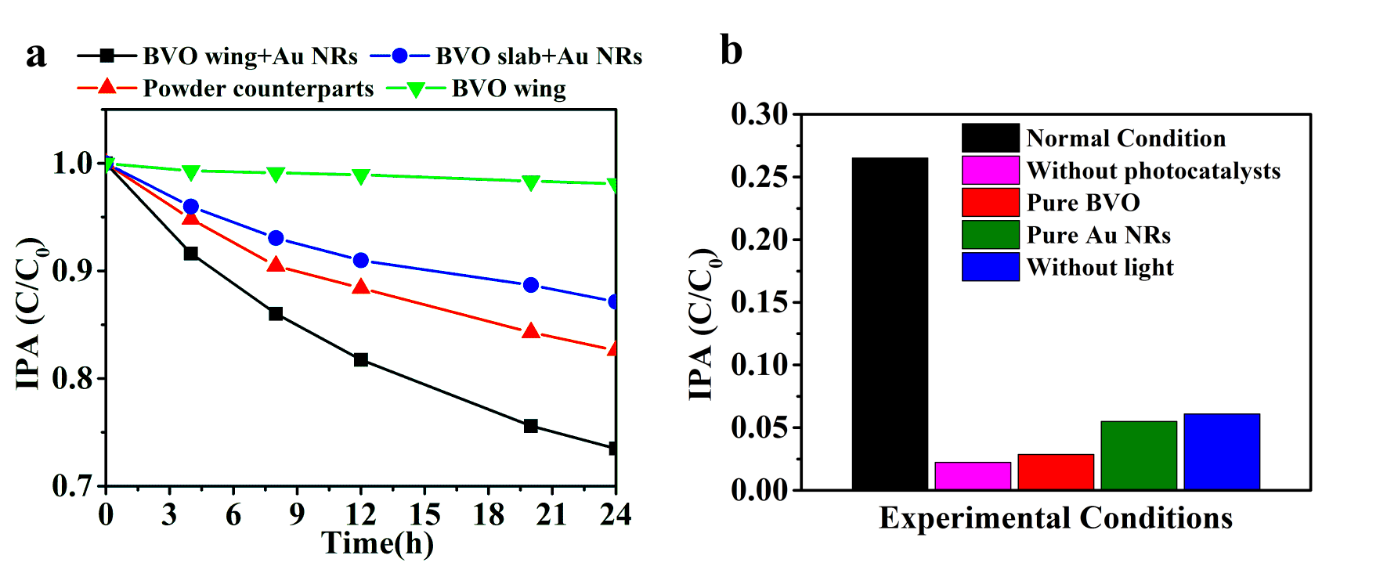


**Supplementary Figure S9. Photocatalytic IPA degradation activities measured by IPA.** (**a**) Percentage of IPA degradation against illumination time over different materials. (**b**) Percentage of IPA degradation after 24 h illumination in different conditions.


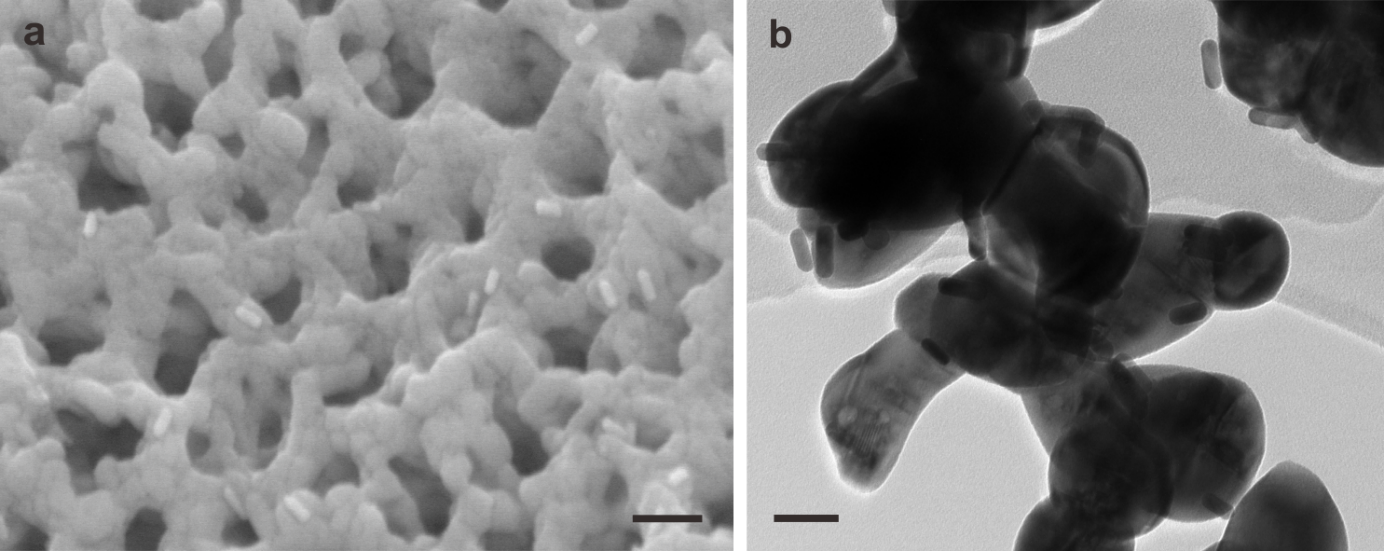


**Supplementary Figure S10.** **SEM and TEM images of Au NRs-loaded BVO wing after three cycles of photocatalytic measurement.** Stability of the photocatalytic system was proved. Scale bars equal 100 nm and 50 nm in (a) and (b), respectively.

**Supplementary Table S1. Dimension figure of different structures.** As the dimension of Papilio nireus structure shank after converting chitin to BVO by the sol-gel method, structural dimensions of original and artificial BVO wing were both listed, indicated as original and artificial respectively.

| **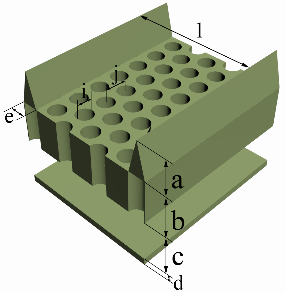Papilio nireus** | 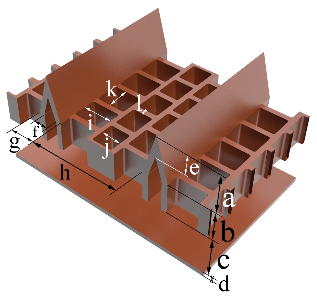**Papilio paris** | 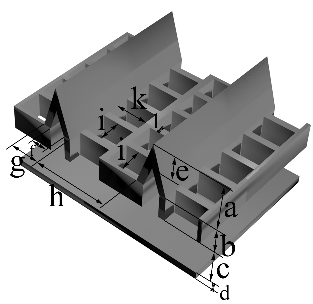**Troides helena’s forewing** | **Troides helena’s underwing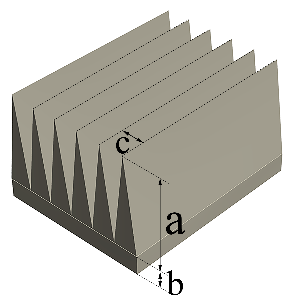** |
| --- | --- | --- | --- |
| **Original:**  **a=745, b=1300, c=1500, d=185, e=240, i=240, j=340, l=2532** | **Original:**  **a=580, b=400, c=660, d=120, e=370, f=130, g=410, h=2420, i=790, j=560, k= 630, l=140** | **Original:**  **a=1760, b=720, c=1730, d=190, e=1100, f=280, g=620, h=1430, i=650, k=470, l=130** | **Original:**  **a=4950, b=2030, e=2000** |
| **Artificial:**  **a=170, b=310, c=350, d=20, e=75, i=75, j=100, l=700** |
